# Polycyclic Aromatic Hydrocarbons–Aromatic DNA Adducts in Cord Blood and Behavior Scores in New York City Children

**DOI:** 10.1289/ehp.1002705

**Published:** 2011-04-12

**Authors:** Frederica P. Perera, Shuang Wang, Julia Vishnevetsky, Bingzhi Zhang, Kathleen J. Cole, Deliang Tang, Virginia Rauh, David H. Phillips

**Affiliations:** 1 Department of Environmental Health Sciences, Mailman School of Public Health, Columbia University, New York, New York, USA; 2 Columbia Center for Children’s Environmental Health, Columbia University, New York, New York, USA; 3 Department of Biostatistics, Mailman School of Public Health, Columbia University, New York, New York, USA; 4 Institute of Cancer Research, Section of Molecular Carcinogenesis, Sutton, Surrey, United Kingdom;; 5 The Heilbrunn Department of Population and Family Health, Columbia University, New York, New York, USA

**Keywords:** cord blood, DNA adducts, neurodevelopment, PAH, ^32^P-postlabeling

## Abstract

Background: Airborne polycyclic aromatic hydrocarbons (PAH) are widespread urban pollutants that can bind to DNA to form PAH–DNA adducts. Prenatal PAH exposure measured by personal monitoring has been linked to cognitive deficits in childhood in a prospective study conducted by the Columbia Center for Children’s Environmental Health.

Objectives: We measured PAH–DNA and other bulky aromatic adducts in umbilical cord white blood cells using the ^32^P-postlabeling assay to determine the association between this molecular dosimeter and behavioral/attention problems in childhood.

Methods: Children born to nonsmoking African-American and Dominican women residing in New York City (NYC) were followed from *in utero* to 7–8 years of age. At two time points before 8 years of age (mean ages, 4.8 years and 7 years), child behavior was assessed using the Child Behavior Checklist (CBCL). To estimate and test the association between adducts and behavioral outcomes, both CBCL continuous raw scores and dichotomized T-scores were analyzed.

Results: Higher cord adducts were associated with higher symptom scores of Anxious/Depressed at 4.8 years and Attention Problems at 4.8 and 7 years, and with Diagnostic and Statistical Manual of Mental Disorders, 4th edition–oriented Anxiety Problems at 4.8 years.

Conclusions: These results suggest that PAH exposure, measured by DNA adducts, may adversely affect child behavior, potentially affecting school performance.

Polycyclic aromatic hydrocarbons (PAH) are a group of pollutants commonly present in ambient air from incomplete combustion of fossil fuels. They are also present in tobacco smoke (Bostrom et al. 2002) and include known carcinogens such as benzo[*a*]pyrene (BaP). After exposure, PAH can exert genotoxic effects, inducing mutations, as well as epigenetic effects (Perera et al. 2009). In addition, PAH bind covalently to DNA to form adducts, a widely used indicator of DNA damage that has been associated with cancer (Bartsch 1996; Poirier and Beland 1992; Rybicki et al. 2004; Stowers and Anderson 1985; Tang et al. 1995, 2002; Veglia et al. 2003).

Laboratory studies exposing experimental animals to PAH during the prenatal and neonatal periods have reported neurodevelopmental and behavioral effects including impairment of memory and ability to learn (Brown et al. 2007; Wormley et al. 2004a) as well as anxiety, and depression-like symptoms in the absence of other overt toxicological effects (Saunders et al. 2006; Takeda et al. 2004; Wormley et al. 2004a; Yokota et al. 2009). In children, anxiety and depression are internalizing problems [problems within the self, rather than externalizing problems, which are conflicts with other people (Achenbach and Rescorla 2001b)] that can affect learning (Emslie 2008; Wood 2006), possibly by interfering with knowledge acquisition (Sanson et al. 1996) or slowing cognitive processing (Emerson et al. 2005), and may precede negative school performance (Horn and Packard 1985). Research in humans has found that anxiety and depression negatively predicted cognitive functioning in a cohort of school-age children (Rapport et al. 2001). In addition, children who scored in the borderline or clinical range of anxiety and depression also scored significantly lower in multiple other domains including attention and processing speed, basic math and spelling skills, and executive function skills (Lundy et al. 2010). Horn and Packard (1985) reported that both internalizing behavior problems and attention-distractibility (an externalizing behavior problem) predicted scholastic achievement during elementary school. Finally, attention problems at school entry have been found to negatively predict math and reading achievement in high school (Breslau et al. 2009).

Molecular epidemiologic studies have provided evidence that the fetus is more susceptible than the adult to the effects of PAH and other toxicants [Anderson et al. 2000; Grandjean and Landrigan 2006; National Research Council (NRC) 1993; Perera et al. 2004; World Health Organization 1986]. In the Columbia Center for Children’s Environmental Health New York City (CCCEH NYC) cohort under study in the present report, prenatal exposure to PAH, as measured by prenatal air monitoring, has been associated with developmental delay at age 3 years (Perera et al. 2006) and reduced IQ at age 5 years (Perera et al. 2009). In addition, higher levels of cord PAH–DNA adducts have been associated with reduced scores on neurocognitive tests, alone or in combination with environmental tobacco smoke (ETS) (Perera et al. 2006, 2007, 2009; Tang et al. 2006).

To date there have been no reports of associations between DNA adducts and child behavior. Here we have assessed the relation between cord PAH/bulky-DNA adducts and behavior problems (especially anxiety/depression and attention problems) at age 4.8 years (range 3.75–5.9 years) and age 7 years (range 6–8 years) using the age-appropriate Child Behavior Checklist (CBCL) (Achenbach and Rescorla 2000, 2001a). The CBCL has been widely used and is sensitive to diverse prenatal environmental exposures (Axtell et al. 2000; Rauh et al. 2006; Robinson et al. 2008; Wasserman et al. 2001).

## Methods

*Sample selection.* A complete description of the CCCEH NYC cohort and study design appears elsewhere (Perera et al. 2003, 2006). Briefly, African-American and Dominican women who resided in Washington Heights, Harlem, or the South Bronx in New York City, USA, were recruited between 1998 and 2003 into a prospective cohort study (Perera et al. 2003). To reduce the potential for confounding, we limited enrollment to women who were in the age range of 18–35 years; non–cigarette smokers; nonusers of other tobacco products or illicit drugs; free of diabetes, hypertension, or known HIV; and who initiated prenatal care by the 20th week of pregnancy. We complied with all applicable requirements of the United States, and the institutional review board of the New York Presbyterian Medical Center approved the study. All women provided written informed consent prior to study initiation and at each visit; children provided assent starting at 7 years of age.

*Personal interview and HOME inventory.* A trained bilingual interviewer administered a 45-min questionnaire during the last trimester of pregnancy to obtain demographic information, residential history, and health and environmental data such as active and passive smoking. ETS exposure was self-reported as having at least one smoker in the home, dichotomized as a yes/no variable (Perera et al. 2003). The questionnaire also elicited information on dietary PAH (consumption of broiled, fried, grilled, or smoked meat), and socioeconomic information related to income and education (Perera et al. 2003). Postnatal maternal interviews were administered in person when the child was 6 months and annually thereafter to determine any changes in residence, exposure to ETS, and other health or environmental conditions. The Home Observation for Measurement of the Environment (HOME) Inventory (Bradley 1994) was used to assess the quality of the home caretaking environment. Self-reported maternal demoralization was measured by the Psychiatric Epidemiology Research Instrument Demoralization Scale (Dohrenwend et al. 1978). We administered the Test of Nonverbal Intelligence-Second Edition (TONI-2), a language-free measure of intelligence (Brown et al. 1990; Caldwell and Bradley 1979) to the mothers at about child age 3 years.

*Biomarkers.* Umbilical cord blood (30–60 mL) was collected at delivery (Perera et al. 2004). The nuclease P1 digestion enhancement procedure of the ^32^P-postlabeling assay was used to analyze bulky/hydrophobic DNA adducts in umbilical cord blood samples having a sufficient yield of DNA (≥ 12 µg). The ^32^P-postlabeling assay is highly sensitive and can be used to detect various DNA adducts with multiple structures (Phillips and Arlt 2007). It can detect adducts in the range of one per 10^8^–10^9^ nucleotides using a 4-µg DNA sample. The bulky/hydrophobic DNA adducts detected by the assay include those formed by PAHs and other genotoxic carcinogens, for example, nitro-PAH and aromatic amines.

In this assay, tissue DNA is degraded enzymatically to mononucleotides; these are then ^32^P-labeled via T4 polynucleotide kinase-catalyzed [^32^P]phosphate transfer from [γ-^32^P]ATP to form 5´-^32^P-labeled 3´,5´-bisphosphate derivatives. The labeled products are resolved by multidirectional thin-layer chromatography and detected and quantified as described (Phillips and Arlt 2007; Randerath et al. 1989). An aliquot (4 µg) of each DNA sample was analyzed on three separate occasions in batches of between 20 and 28 samples. For each assay, a positive control consisting of DNA modified with BaP diol-epoxide was also analyzed.

Lead was measured at the Centers for Disease Control and Prevention (CDC) in a subset of cord bloods (*n* = 153) using inductively coupled plasma mass spectrometry (CDC/Division of Laboratory Science 2003). To adjust for postnatal exposure to PAH, urinary PAH metabolites were measured in a subset of children at 5 years of age (*n* = 102). Urine was analyzed, as DNA from blood samples was not available for this assay and urinary PAH metabolites are a well-validated internal dosimeter for these compounds. The CDC measured a suite of 22 PAH metabolites, using enzymatic deconjugation, followed by automated liquid–liquid extraction and quantified by gas chromatography/isotope dilution high-resolution mass spectrometry (Li et al. 2008).

*Behavioral outcomes.* Research workers trained in neurodevelopmental testing administered the CBCL to the mothers, using the 99-item CBCL for children < 6 years of age (Achenbach and Rescorla 2000) and the 118-item CBCL for children ≥ 6 years (Achenbach and Rescorla 2001a). The syndrome scores were computed for each domain of *a priori* interest (Anxious/Depressed and Attention Problems) by summing the scores on the specific items, which yields a raw score. This raw score can be converted to a standardized T-score. We report results from two versions of the CBCL administered at two different ages; the two versions have different numbers of items and distributions of scores. T-scores were generated by the CBCL software according to the procedure of Abramowitz and Stegun (1968). A T-score of 50 is assigned to children with percentiles of raw scores ≤ 50 based on a reference population (Achenbach and Rescorla 2000), whereas those with percentiles of raw scores > 50 are assigned the actual T-score.

The CBCL also yields scales derived from the Diagnostic and Statistical Manual of Mental Disorders, 4th edition (DSM-IV) (American Psychiatric Association 2000), that are intended to approximate clinical diagnoses. The DSM-IV scores are dichotomized using a borderline or clinical cut point corresponding to the 93rd percentile for each domain (Achenbach and Rescorla 2000). Based on our *a priori* hypothesis, we focused on the DSM-oriented Anxiety Problems in our analysis.

*Statistical methods.* Cord DNA adducts (range 0.2–24.8 adducts/10^8^ nucleotides) were dichotomized at the upper quartile (2.7 adducts/10^8^ nucleotides) of the 461 children with data on DNA adducts and maternal prenatal questionnaire data. Among the 215 children in the present analysis, we identified 58 and 157 subjects as high exposed and low exposed, respectively. Although the dichotomized adduct variable is our independent variable of choice, we have also used logarithm-transformed adducts as a continuous variable. Covariates were selected based on whether they were significant contributors to the model (here at *p* ≤ 0.1) for at least one of the outcomes. Although ethnicity (African American or Dominican) was not a significant covariate in the model, it was included because it is highly correlated with the CBCL scores and there is a significant difference in ethnicity between the subjects included and those not included. Covariates in the models included ETS exposure during pregnancy, sex of child, gestational age of the child, intelligence of mother as measured by TONI-2, completed years of education of the mother before birth of the child, age of child at assessment in months, quality of the early home caretaking environment measured at 3 years of age, and season of the last trimester (either during the heating season from 1 October to 30 April, when ambient PAH levels are higher, or not). Gestational age was based on medical record data for almost all subjects. Covariates are defined in Table 1.

**Table 1:** Characteristics of the sample (mean ± SD or %).

Subjects in the analysis (*n* = 215)	Subjects not included*a *(*n* = 246)		
	Variable	Value	*n*
Cord ^32^P adducts*b*		2.45 ± 2.43		2.32 ± 2.31		246
Prenatal ETS*c*		36.28		34.15		246
Female*d*		59.53		47.56		246
Gestational age*e*		39.68 ± 1.49		39.67 ± 1.34		246
Maternal demoralization score*f*		1.19 ± 0.62		1.16 ± 0.67		219
Maternal education*g*		11.84 ± 2.18		11.85 ± 2.27		246
Maternal TONI score*h*		20.77 ± 8.70		19.89 ± 8.50		144
Home environment*i*		39.98 ± 6.26		38.62 ± 6.52		129
Ethnicity (AA%)*d,j*		44.65		30.08		246
Heating season*k*		53.95		59.57		235
**a**Some subjects were not included because of missing CBCL or information on at least one covariate. **b**The ^32^P-postlabeling assay was used to measure adducts. Units are adducts/10^8^ nucleotides. **c**Prenatal ETS exposure in the home. **d**Significantly different between the two groups based on Pearson’s chi-square test. **e**Gestational age in weeks. **f**Maternal demoralization during pregnancy. **g**Maternal education in years of school. **h**Nonverbal intelligence measured by the TONI. **i**HOME Inventory as a measure of the home caretaking environment. **j**Percentage African American; the remainder are Dominican. **k**Third trimester in heating season.						

With respect to CBCL syndrome scores, both the continuous raw scores and the dichotomized T-scores were analyzed. Because the raw scores of each domain of interest are counts data that sum the scores on the specific items within each syndrome scale, we applied the Poisson model on the raw scores for the syndromes. We further analyzed the syndrome T-scores dichotomized at 65 (the cutoff for scores for the borderline and clinical range) (Achenbach and Rescorla 2000) using a logistic model. We used logistic regressions for analysis of dichotomized DSM-oriented Anxiety Problems (cutoff at 93rd percentile for the borderline and clinical range).

To estimate associations between PAH and behavior outcomes at different ages, two separate analyses were done on CBCL measures at (mean) 4.8 years (*n* = 96) and (mean) 7 years (*n* = 205). Note that 86 children had measures at both time points.

To check for the possible confounding effect of postnatal PAH exposure, we fit a separate model with CBCL measurements of children at 7 years adjusting for PAH metabolites in child urine collected at 5 years of age. Because of incomplete data on postnatal PAH exposure, the model with postnatal PAHs had a smaller sample size (*n* = 100 at age 7 years). Moreover, to check the possible confounding effect of postnatal ETS exposure, we fit separate models adjusting for postnatal ETS exposure measured at 2 years of age. Similarly, because of incomplete data on postnatal ETS exposure, the separate models had smaller sample sizes (*n* = 85 at age 4.8 years and *n* = 182 at age 7 years). We also fit a model that included an interaction term between adducts and sex of the child to check for possible interactions.

All effect estimates, 95% confidence intervals (CIs), and *p*-values (α set at 0.05) were generated using SAS (version 9.1.0.3; SAS Institute Inc., Cary, NC, USA).

## Results

Four hundred sixty-one children had data on DNA adducts in cord blood as well as prenatal questionnaire data. Missing adduct data resulted from failure to collect cord blood or insufficient amounts of DNA available for the assay. Two hundred fifty-two of the children had CBCL data obtained at least once at (average) ages 4.8 years or 7 years (ranges, 3.75–5.9 and 6–8 years, respectively). The subset included in the present analysis was composed of 215 of these mother–child pairs who also had available data on all explanatory or potential confounding variables of interest. The remainder of the 461 study participants either had a CBCL measurement completed outside of the identified time period (*n* = 109), did not have a CBCL measurement at all (*n* = 100), or were missing data on key covariates (*n* = 37).

CCCEH NYC participants included in the present study (*n* = 215) were similar to participants with adduct data who were not included because of missing CBCL or covariate data (*n* = 246) with respect to level of cord DNA adducts and ETS exposure, gestational age, maternal education, self-reported maternal demoralization, the quality of the home caretaking environment (HOME), and season of the last trimester (Table 1). However, children included in the present analysis were more likely to be female and African American than those not included (Table 1). We also compared the 461 children with cord adducts measured by ^32^P-postlabeling with children lacking adduct data and found that they differed significantly only with regard to gestational age (39.68 weeks vs. 39.26 weeks, *p* = 0.0006).

Table 2 provides the distribution of CBCL scores. Both raw scores and T-scores are highly right skewed. As expected, the mean scores in our study are similar to mean scores of the normative sample of children who have not been referred to mental health services or special education (Achenbach and Rescorla 2001b, 2001c). Correlation coefficients between the syndrome scores of Anxious/Depressed and Attention Problems ranged from 0.37 to 0.57.

**Table 2:** Distribution of selected outcomes in the CCCEH NYC cohort.

Borderline or clinical range [*n* (%)]*b*
	Score range			Mean of scores		
	Mean age (range)	*n*	Outcome	T-score*a*	Raw score	T-score	Raw score
4.8 years (3.75–5.91 years)		96*c*		Anxious/depressed		50–87		0–13		54.80		3.01		10 (10.42)
				Attention problems		50–73		0–8		54.35		2.52		10 (10.42)
				Anxiety problems (DSM)				0–16				3.63		10 (10.42)
7 years (6–8 years)		205*c*		Anxious/depressed		50–82		0–17		53.72		2.77		17 (8.29)
				Attention problems		50–83		0–16		54.60		3.38		14 (6.83)
				Anxiety problems (DSM)				0–9				1.56		21 (10.24)
**a**The T-score is truncated (Petersen et al. 1993); that is, a score of 50 is assigned to those with percentiles of raw scores ≤ 50 based on a reference population (Achenbach and Rescorla 2001b). **b**The syndrome T-scores were dichotomized at T = 65 as the cutoff for the borderline and clinical range; the DSM-oriented anxiety problem scale was dichotomized at 93rd percentile for the borderline and clinical range. **c**Eighty-six children had been assessed via the CBCL at both age time points.														

In simple Poisson models without adjustment for covariates (data not shown), the children with higher levels of cord DNA adducts had higher scores, consistent with increased Anxious/Depressed and Attention Problems at (mean) 4.8 years and at (mean) 7 years, relative to children with lower cord DNA adduct levels. In the full Poisson model after adjusting for possible confounders, higher (vs. lower) cord adducts were associated with the syndrome score of Anxious/Depressed (β = 0.34, 95% CI, 0.04–0.64, *p* = 0.026) and Attention Problems (β = 0.38, 95% CI, 0.06–0.69, *p* = 0.018) at (mean) 4.8 years and with Attention Problems at (mean) 7 years (β = 0.22, 95% CI, 0.06 to 0.38, *p* = 0.009) (Table 3). The beta coefficient 0.34 of the adduct high/low variable on symptoms of Anxious/Depressed suggests that subjects in the upper quartile of adducts have about a 40% [exp(0.34) = 1.4] increase in symptom scores compared with subjects with adduct levels below the 75th percentile; that is, for subjects with mean CBCL Anxious/Depressed scores of 3, those in the upper quartile will have scores 1.2 points higher, on average, than subjects who have lower adduct levels. After Bonferroni correction, the associations between cord DNA adducts and CBCL outcomes did not remain significant. However, the Bonferroni correction is considered to be overly conservative in initial studies such as this (Wacholder et al. 2004)

**Table 3:** Associations between DNA adducts in cord blood and CBCL syndrome and DSM-oriented outcomes.*a*

CBCL: Anxious/depressed							CBCL: Attention problems							DSM: Anxiety problems		
Poisson raw			Logistic dichotomized T			Poisson raw			Logistic dichotomized T			Logistic model		
Exposure	β (95% CI)	*p*-Value	OR (95% CI)	*p*-Value	β (95% CI)	*p*-Value	OR (95% CI)	*p*-Value	OR (95% CI)	*p*-Value
Cord ^32^P adducts, age 4.8 years*b,c*		0.34 (0.04–0.64)		0.026*		8.14 (1.21–54.94)		0.031*		0.38 (0.06–0.69)		0.018*		5.66 (0.64–50.05)		0.119		8.30 (1.13–60.71)		0.037*
Cord ^32^P adducts, age 7 years*b,d*		–0.03 (–0.22 to 0.16)		0.773		1.42 (0.45–4.46)		0.544		0.22 (0.06–0.38)		0.009*		3.30 (1.22–12.54)		0.022*		1.26 (0.42–3.82)		0.683
**a**The model includes prenatal ETS, sex of child, gestational age, maternal IQ, HOME inventory, maternal education, ethnicity, prenatal demoralization, age at assessment, and heating season as covariates. **b**Adducts were dichotomized at upper quartile. **c**Range: 3.75–5.91 years, *n* = 96, with 80 children classified as low exposure and 16 children classified as high exposure. **d**Range: 6–8 years,* n* = 205, with 149 children classified as low exposure and 56 children classified as high exposure. *p = < 0.05.																				

After controlling for postnatal exposure to ETS at 24 months, the association between DNA adducts and the syndrome score of Attention Problems became more significant at 4.8 years with a higher beta (β = 0.45, *p* = 0.012, *n* = 85) and at age 7 years (β = 0.25, *p* = 0.006, *n* = 182) and remained significant for the syndrome score of Anxious/Depressed (β = 0.37, *p* = 0.031, *n* = 85) at 4.8 years of age. After controlling for PAH metabolites in child urine collected at 5 years of age, higher cord adducts were significantly associated with the syndrome score of Attention Problems at 7 years (β = 0.28, *p* = 0.028, *n* = 100). These latter analyses involved limited numbers of subjects, so power was reduced accordingly.

As a further analysis, we used logistic regression on T-scores dichotomized at 65 for the syndrome scales (Table 3). The results were significant for the syndromes of Attention Problems at 7 years [odds ratio (OR) = 3.30, 95% CI, 1.21–12.54] and Anxious/Depressed at 4.8 years (OR = 8.14, 95% CI, 1.21–54.94), showing that higher cord adducts were associated with increased likelihood of borderline or clinical classification on Anxious/Depressed and Attention Problems. We also used the logistic model on DSM-oriented Anxiety Problems (Table 3). The results show that higher cord adducts were associated with increased likelihood of borderline or clinical classification on the DSM-oriented Anxiety Problem Scale (OR = 8.30, 95% CI, 1.13–60.71) at 4.8 years of age.

With continuous adducts used as the main predictor, the results were consistent with those using the dichotomized cord adduct variable for Attention Problems at 7 years of age; that is, they had the same direction of the association and were statistically significant (β = 0.19, *p* < 0.001); however, the adducts were no longer significant for age 4.8 years, nor were they significant with the syndrome of Anxious/Depressed at either time point. The use of dichotomized cord adducts is preferable because dichotomization is less vulnerable to errors in measurement, does not materially lose information, and permits comparison of the most highly exposed children with those who had lower exposure.

Correlations between cord adducts and ETS and dietary PAHs, respectively, were not significant using Spearman rank-order correlation (*r* = 0.05, *p* = 0.43) and Pearson correlation (*r* = –0.04, *p* = 0.55). The correlation between adducts and lead was also not significant in the limited subset with both measurements (by Pearson correlation: *r* = 0.06, *p* = 0.44, *n* = 153). Dietary PAHs and lead were not associated with outcomes at α = 0.1 and were therefore not included in the models. In addition, as stated above, we had the data for only a subset of the sample.

## Discussion

Previous results from this cohort study have provided evidence that prenatal exposure to PAH air pollutants during pregnancy is a risk factor for developmental delay at 3 years of age, as measured by the Bayley Scales of Infant Development (Perera et al. 2006), and for reduced child IQ at 5 years. To our knowledge, there have been no prior molecular epidemiologic studies of the role of prenatal DNA adducts in child behavior. The present analysis, using bulky/hydrophobic DNA adducts detected by ^32^P-postlabeling analysis as a molecular dosimeter for PAHs and other combustion-related pollutants, suggests an adverse effect of those exposures on child behavioral problems that potentially could affect cognitive test scores and ability to learn. We did not observe clear differences in associations between DNA adducts on child behavioral problems among boys and girls (adduct–sex interactions were not significant in any syndrome scores at either age; all *p*-values > 0.6).

The associations between DNA adducts and syndromes of Attention Problems and Anxious/Depressed observed in this New York City population are consistent with the experimental data on PAHs (Saunders et al. 2006; Takeda et al. 2004; Wormley et al. 2004a; Yokota et al. 2009). Prenatal treatment of rats with BaP impaired memory and ability to learn, consistent with alterations in the expression profile of key genes involved in long-term potentiation (Hood et al. 2000; Wormley et al. 2004b). Fetal BaP exposure also influenced the expression of nuclear transcription factors that mediate the onset of neuronal cell differentiation, suggesting that there may be widespread effects of this agent in the developing brain, ultimately contributing to neurobehavioral impairment (Hood et al. 2000).

The mechanisms by which PAHs might affect the developing brain are not fully known. Fetal toxicity may be caused by endocrine disruption (Archibong et al. 2002; Bui et al. 1986; Takeda et al. 2004), binding to receptors for placental growth factors resulting in decreased exchange of oxygen and nutrients (Dejmek et al. 2000), binding to the human Ah receptor to induce P450 enzymes (Manchester et al. 1987), DNA damage resulting in activation of apoptotic pathways (Meyn 1995; Nicol et al. 1995; Wood and Youle 1995), epigenetic effects (Wilson and Jones 1983), or oxidative stress due to inhibition of the brain antioxidant scavenging system (Lundqvist et al. 2006). The prenatal period is highly sensitive to neurotoxic effects of environmental contaminants (Nijland et al. 2008; Rodier 2004).

In our cohort, DNA adducts were associated with CBCL test scores consistent with increased attention problems at 4.8 and 7 years of age and increased anxiety and depression scores at 4.8 years of age. Attention problems at 6 years of age have been associated with achievement in high school; this may be due to children never fully grasping elementary concepts, making comprehending more complex ideas very challenging (Breslau et al. 2009). Prior research has shown that internalizing behavioral problems may lead to a wide range of learning deficits in areas ranging from problem-solving tasks to verbal memory to intellectual function (Emerson et al. 2005; Gunther et al. 2004; Lundy et al. 2010). Finally, attentional and internalizing problems are worrisome outside the academic domain as well. Attention deficits and anxious and depressive behaviors are linked to difficulties in peer relationships and other social functioning (Alfano and Gamble 2009; Esbjørn et al. 2010; Ladd 1999; Swords et al. 2011). The children in our cohort are being followed to 12 years of age; therefore, subsequent testing will provide a picture of the longer-term developmental outcomes of children in the cohort.

The strengths of the analysis include our ability to account for a number of factors other than PAH exposure that are known to affect child behaviors. We were able to draw upon individual prenatal exposure data from personal monitoring, biomarker data, and extensive medical record and questionnaire data. A limitation of this research is our relatively small sample size of the subjects in the analysis. This attrition is attributable to the exclusion of subjects who are missing complete covariate data or testing results. There is a possible effect of selection bias, because there is a difference in the sex and ethnicity distribution of the included and excluded subjects. Another limitation is the reduced number of children with lead measurements, limiting interpretation of analyses controlling for lead exposure. Although lead was not found to be significantly correlated with DNA adducts, CBCL, or potential confounders in the present data set, it is possible that we lack the necessary sample size to test the relationships adequately.

## Conclusion

In conclusion, this study suggests that prenatal exposure to PAHs at levels encountered in the air of New York City may adversely affect child behavior. The results are of potential concern, because attention problems and anxiety and depression may affect subsequent academic performance as well as peer relationships and other aspects of societal functioning (Emslie 2008; Esbjørn et al. 2010; Swords et al. 2011; Wood 2006). PAHs are widespread in urban environments in the United States and worldwide largely as a result of fossil fuel combustion for energy production and transportation. Fortunately, it is possible to reduce airborne PAH concentrations through currently available pollution controls, energy efficiency, alternative energy sources (Wong et al. 2004), and regulatory intervention to remove or control polluting sources (Millman et al. 2008).
